# Performance of ChatGPT in classifying periodontitis according to the 2018 classification of periodontal diseases

**DOI:** 10.1007/s00784-024-05799-9

**Published:** 2024-06-29

**Authors:** Zeynep Tastan Eroglu, Osman Babayigit, Dilek Ozkan Sen, Fatma Ucan Yarkac

**Affiliations:** Department of Periodontology, Necmettin Erbakan University Faculty of Dentistry, Beyşehir Caddesi, Bağlarbaşı Sk., 42090 Meram, Konya, Turkey

**Keywords:** Classification, Diagnosis, Periodontal diseases, Artificial intelligence, Language processing

## Abstract

**Objectives:**

This study assessed the ability of ChatGPT, an artificial intelligence(AI) language model, to determine the stage, grade, and extent of periodontitis based on the 2018 classification.

**Materials and methods:**

This study used baseline digital data of 200 untreated periodontitis patients to compare standardized reference diagnoses (RDs) with ChatGPT findings and determine the best criteria for assessing stage and grade. RDs were provided by four experts who examined each case. Standardized texts containing the relevant information for each situation were constructed to query ChatGPT. RDs were compared to ChatGPT's responses. Variables influencing the responses of ChatGPT were evaluated.

**Results:**

ChatGPT successfully identified the periodontitis stage, grade, and extent in 59.5%, 50.5%, and 84.0% of cases, respectively. Cohen’s kappa values for stage, grade and extent were respectively 0.447, 0.284, and 0.652. A multiple correspondence analysis showed high variance between ChatGPT’s staging and the variables affecting the stage (64.08%) and low variance between ChatGPT’s grading and the variables affecting the grade (42.71%).

**Conclusions:**

The present performance of ChatGPT in the classification of periodontitis exhibited a reasonable level. However, it is expected that additional improvements would increase its effectiveness and broaden its range of functionalities (NCT05926999).

**Clinical relevance:**

Despite ChatGPT's current limitations in accurately classifying periodontitis, it is important to note that the model has not been specifically trained for this task. However, it is expected that with additional improvements, the effectiveness and capabilities of ChatGPT might be enhanced.

## Introduction

A periodontal disease classification serves as a diagnostic tool for clinicians to identify periodontal conditions. A classification of periodontal diseases has been used and improved since the 1980s. A new classification for periodontal and peri-implant diseases and conditions was introduced in 2018 and remains in use today [1]. Significant differences between this new classification and the 1999 classification [2] include novel disease forms and categorization, case definitions, and clinical criteria for each periodontal and peri-implant condition. This detailed knowledge is provided in a consensus reports [3, 4]. Various diagnostic tools have been developed to aid clinicians in the decision-making process based on the 2018 periodontal classification. Additionally, simple flowcharts and other decision-making algorithms have been proposed [5, 6]. For instance, the Italian Society of Periodontology and Implantology recently created a software application [7].

ChatGPT (https://chat.openai.com/) is an implementation of the Generative Pre-trained Transformer 3 (GPT-3.5) language model developed by OpenAI, which is freely available to the public [8]. GPT-3.5 is a highly expansive neural network–based natural language processing (NLP) model currently one of the largest in existence. With training on 175 billion parameters, its primary purpose is to generate text that closely resembles human language. Acting as a versatile chatbot, GPT-3.5 is capable of performing diverse NLP tasks, such as language translation, summarization, and answering questions [9]. Furthermore, it can offer alternative differential diagnostic support [8]. Clinicians can obtain a list of potential diagnoses and advice on subsequent management choices by providing the model with case details [9]. Better differential diagnostic assistance systems may be created by considering the strengths and weaknesses of such models. To explore this possibility, this preliminary study aimed to evaluate the ability of ChatGPT to determine the stage, grade, and extent of periodontitis according to the 2018 classification when provided with rich case descriptions, understanding the capabilities and limitations of this tool.

## Materials and methods

### Study design

This research was based on an analysis of the baseline digital records and subsequent stage, extent, and grade characterizations of 200 untreated patients diagnosed with periodontitis. All cases were evaluated by four examiners to obtain gold standardized diagnoses. The information used to determine the stage, grade, and extent of periodontitis was inputted directly to ChatGPT, followed by the query, “What is the stage, grade, and extent of periodontitis?” The chatbot’s replies were then compared with the standardized diagnoses.

### Ethical considerations

From March 2023 to July 2023, baseline clinical and radiographic documentation of patients with periodontitis was collected in the context of regular visits to the Periodontology Department of the Faculty of Dentistry. The patients’ data were anonymized. All participants provided written consent for the use of their data for training and research. This study adhered to the 2013 revision of the 1975 Declaration of Helsinki and was approved by the Ethics Committee for Non-pharmaceutical and Medical Device Clinical Research of the Faculty of Dentistry of Necmettin Erbakan University (Decision no. 2023/303). The study protocol was registered on ClinicalTrials.gov (NCT05926999).

### Selection criteria for periodontitis case

**Fig. 1:**
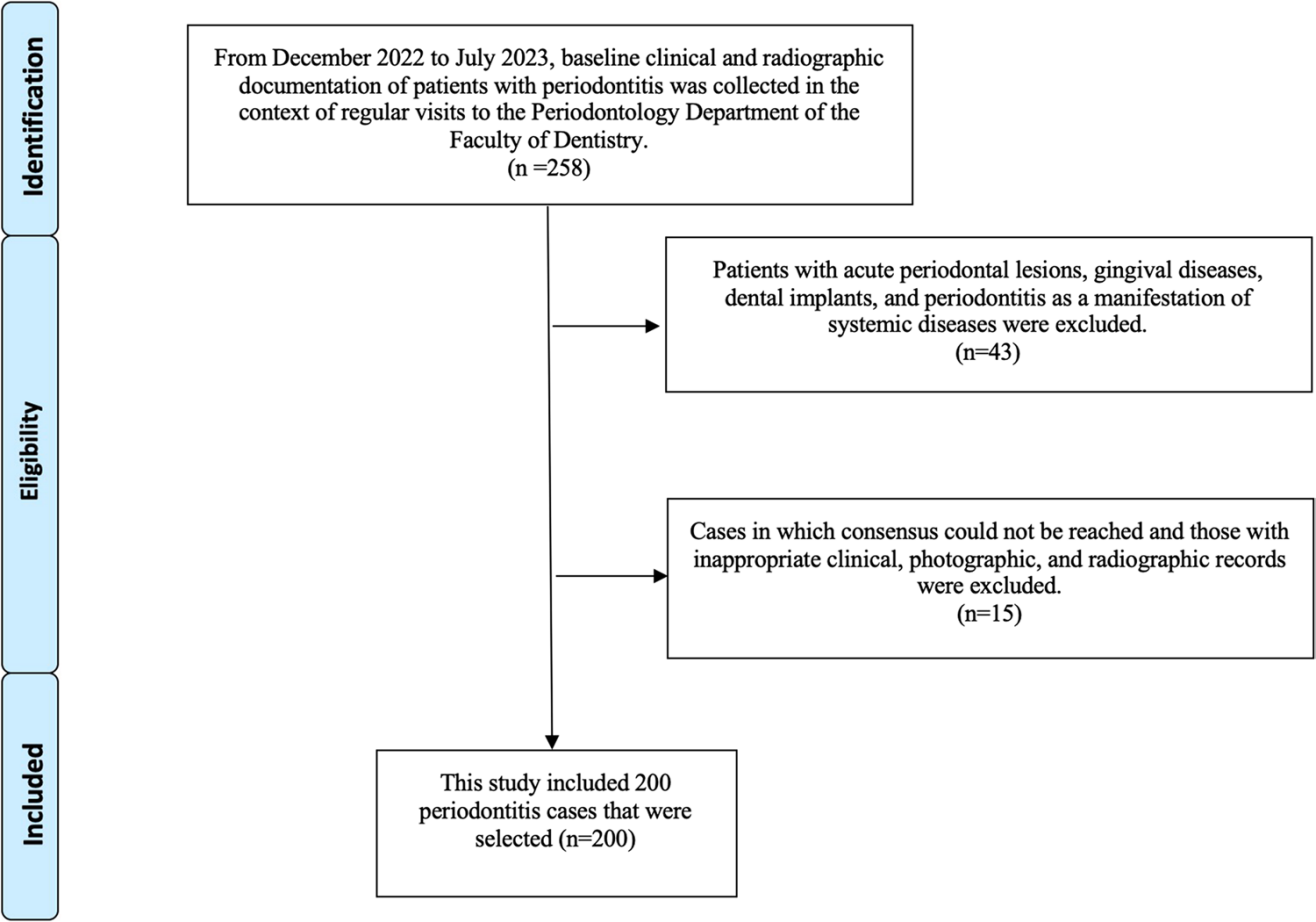
Flow chart of this study

258 periodontitis cases were selected from the archive of patients of the Periodontology Department at Necmettin Erbakan University (Konya, Turkey). Patients with acute periodontal lesions, gingival diseases, dental implants, and periodontitis as a manifestation of systemic diseases were excluded (n = 43). The four experts, who are responsible for providing the standardized reference diagnoses (RDs) and are described in detail below, reviewed and discussed the cases collaboratively, reaching a consensus through open discussion. Cases in which consensus could not be reached and those with inappropriate clinical, photographic, and radiographic records were excluded from the study (n = 15). As a result, the experts provided consistent diagnoses for the remaining cases (n = 200), which were considered RDs for the respective cases (Fig. 1). This collaborative approach ensured that the diagnoses were as accurate and standardized as possible, aligning with the 2017 World Workshop criteria [10].

### Preparation of documentation for the periodontitis cases

Each case description included a comprehensive summary of the patient’s medical and dental history, intraoral photographs, a panoramic radiograph, a complete set of periapical radiographs, and periodontal charting that encompassed various clinical measures related to periodontal health. The measures were plaque scores [11], probing depth, bleeding on probing, clinical attachment loss (CAL), furcation involvement (FI) [12], and tooth mobility [13]. The dental records of each patient included information regarding various aspects of their oral health, such as gingival bleeding, tooth mobility, dentin hypersensitivity, halitosis, family history of periodontitis, utilization of inter-dental oral hygiene devices, usage of mouthwashes, presence of para-functional habits, chewing dysfunction, tooth migration, prior orthodontic treatment, previous periodontal treatment, and previous prosthetic treatment. Moreover, the last dental examination and professional oral hygiene procedure (≤ 1 year, > 1 year or > 3 year) and the number of teeth loss due to periodontitis (0, ≤ 4 or ≥ 5) were reported [14]. Each patient’s medical history contained details on pertinent medical issues, including glycemic management and cigarette use.

**Fig. 2:**
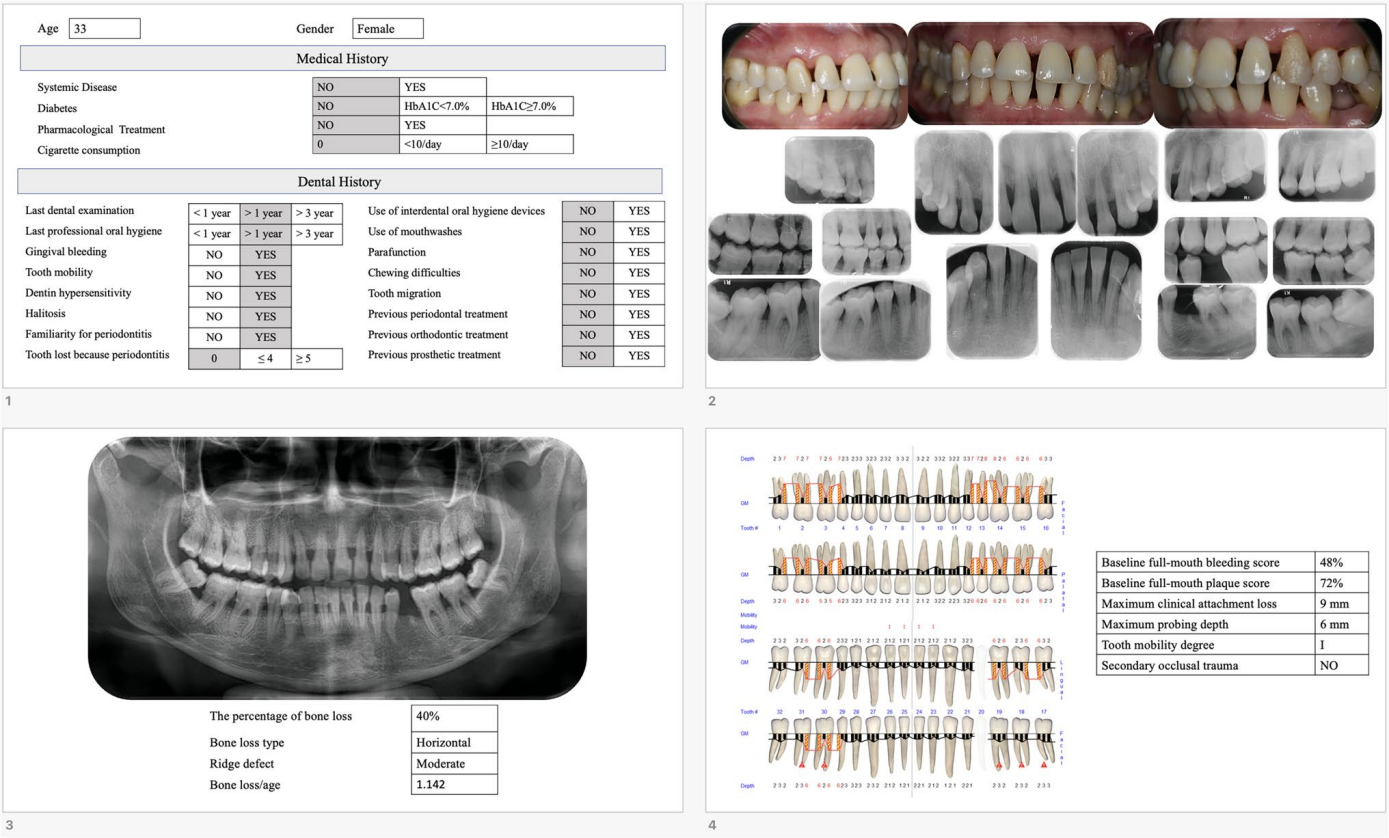
Representative example of documentation provided for each case

The entire documentation was compiled into a presentation file (Fig. 2). This presentation file was evaluated together by four experts.

### Providing of standardized reference diagnoses

Four experienced periodontists employed as full-time faculty members (Z.T.E., O.B., D.O.S., and F.U.Y.) evaluated the cases using the prepared presentations. The four experts had gone over the 2018 periodontal classification consensus reports multiple times and had been using the classification for clinical diagnoses for at least four years. The experts utilized the 2017 World Workshop on the Classification of Periodontal and Peri-Implant Diseases and Conditions as the basis for diagnosing cases [4, 10]. The extent, which refers to the overall severity and complexity of the case, was described subsequent to determining of the staging (For each stage, describe extent as localized [< 30% of teeth involved], generalized, or molar/incisor pattern.). Evident hopeless teeth were included when calculating the number of teeth lost because of periodontitis for distinguishing between Stages III and IV periodontitis. Teeth in which the attachment loss approximates the apex of the root circumferentially and in combination with a high degree of tooth hyper- mobility (degree III) were described as evident hopeless teeth (also termed irrational to treat) [15].

Standardized digital periapical and panoramic radiographs (Veraviewepocs 3D F40, Morita, Japan) were employed to assess bone loss using computer software (Turcasoft Dent, Samsun, Turkey). Following the landmarks identified by Nibali et al. [16], the cemento-enamel junction (CEJ), the radiographic apex, and the bottom of the alveolar bone crest (bone level, BL) were established. Measurements were taken between the CEJ and the apex (CEJ-apex) and the CEJ and BL (CEJ-BL) at the most severely affected tooth to the nearest millimeter (mm). The percentage of radiographic bone loss (RBL) was then calculated by dividing CEJ-BL by CEJ-apex [16, 17]. When the CEJ was not visible due to prosthodontic restorations, the restoration margin was used as a reference point.

All radiographs were reviewed by the experts and re-evaluated until consensus was reached. For cases with radiographs available from five years prior, bone loss over the past five years was measured in millimeters. When previous radiographs were not available, the percentage of RBL was calculated as described [10]. Both methods were applied to the most severely affected tooth, excluding bone loss around third molars and the distal aspects of second molars. If the distance between the CEJ and the alveolar bone level was less than 2 mm, it was classified as no bone loss, irrespective of the RBL [18].

The four experts assessed the case phenotype in addition to RBL to determine indirect evidence of progression. This evaluation, as delineated by Tonetti et al. [10], was classified into three categories: heavy biofilm deposits with low levels of destruction, where significant biofilm accumulation is present but associated tissue destruction is minimal; destruction commensurate with biofilm deposits, where the extent of tissue destruction is proportionate to the amount of biofilm present; and destruction that exceeds expectations given biofilm deposits, where observed tissue destruction surpasses what would be anticipated based on biofilm accumulation alone.

### Staging, grading, and determining the extent of periodontitis using ChatGPT

The performance of ChatGPT in staging, grading, and determining the extent of periodontitis was evaluated using case descriptions. Since ChatGPT is a language model and cannot use images, the patients’ radiographs were evaluated by the experts. The extent and rate of bone loss were measured and converted to numerical data that ChatGPT could use. Standardized texts containing the information used to determine the stage, grade, and extent of periodontitis for each case were created as follows:

#### Staging

- Age and gender
- Maximum CAL in the interproximal area
- RBL
- The number of teeth lost due to periodontitis
- Maximum probing depth (PD)
- Bone loss type
- FI according to the Hamp classification [12]
- Presence of chewing dysfunction
- Second-degree and above-tooth mobility
- Presence of ridge defects
- Number of teeth in occlusion

#### Determining the extent and distribution of periodontitis

Periodontitis coverage.

#### Grading

- Bone loss in the previous five years (If data is accessible)
- Ratio of RBL to age (RBL/A) in the most affected area
- Phenotype of destruction: heavy biofilm deposits with low levels of destruction (HBLD), destruction commensurate with biofilm deposits (DCB), or destruction exceeding expectations given the amount of biofilm deposits (DEB)
- Smoking status and number of cigarettes per day
- Diabetic status and hemoglobin A1c (HbA1c) levels

**Fig. 3:**
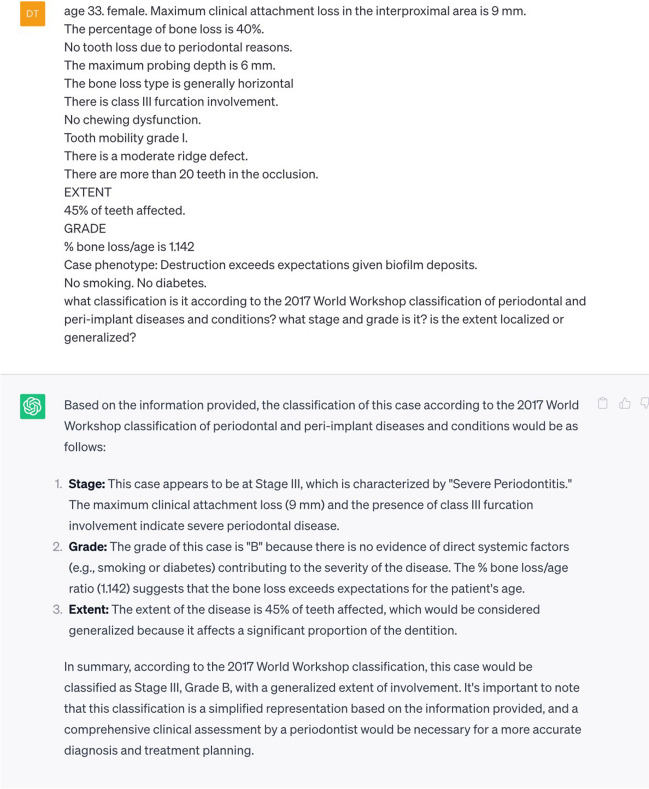
Example of the input text fed into the ChatGPT model with the corresponding output

For the purposes of this study, a ChatGPT account was created in September, 2023. The current ChatGPT version was used. To minimize the impact of prior responses, a new chat window was used for each case. A standardized text was created for every case in the ChatGPT query. It was then asked, “What is the stage, grade, and extent of periodontitis?”. The responses were recorded for later analysis (Fig. 3).

### Outcomes

The primary outcome was the level of agreement between the RDs and the results obtained from ChatGPT. The secondary outcome was the effectiveness of each variable inputted to ChatGPT in determining the periodontitis stage and grade.

### Statistical analysis

Categorical variables were expressed as frequencies and percentages, while numerical variables were expressed as means and standard deviations. Cohen’s kappa values were used to assess agreement in terms of periodontitis grade, stage, and extent. A multiple correspondence analysis of the respective scores was performed using R 4.2.2 software to evaluate the agreement between the RDs and ChatGPT’s responses in terms of CAL, PD, RBL and teeth loss due to periodontitis for stage and RBL/A, the phenotype of destruction, smoking status, and diabetes status for grade. Following Landis and Koch [19], the kappa values were interpreted as follows: ≤ 0 = poor agreement; 0–0.2 = slight agreement; 0.21–0.4 = fair agreement; 0.41–0.6 = moderate agreement; 0.61–0.8 = substantial agreement; and 0.81–1 = almost perfect agreement.

### Sample size

We calculated that a sample of 200 subjects was required to predict a kappa value of 0.4 for inter-rater agreement of ChatGPT and RD ratings. We used the method presented in Rotondi MA, Donner A. [20]. Based on previous data from our clinic, with predetermined stage I, II, III and IV rates of 13%, 39%, 39%, 9%, respectively. We used a length of 0.2 with a 95% confidence interval and a Type 1 error rate of 5%.

## Results

### Descriptive characteristics of the periodontitis cases

The flow chart of this study is shown in Fig. 3. The 200 cases analyzed in this study comprised the full periodontitis spectrum. The descriptive characteristics of the periodontitis cases are shown in Table 1. According to the analysis conducted by ChatGPT, out of a total of 200 instances, 78 cases (39%) were classified as stage I, 44 cases (22%) were classified as stage II, 74 cases (37%) were classified as stage III, and 4 cases (2%) were classified as stage IV. In relation to the grading system, 91 cases (46%) were classified as grade A, 85 cases (43%) were classified as grade B, and 24 cases (12%) were classified as grade C. A total of 136 cases (68%) were classified as localized, while the remaining 64 cases (32%) were categorized as generalized.

**Table 1:** Descriptive characteristics of the two hundred periodontitis cases

Characteristics	Frequency (n)	Percentage (%)
Age		
Years; mean ± SD	39.83 ± 10.24	
Gender		
Males	75	38%
Females	125	63%
Stage		
I	45	23%
II	64	32%
III	57	29%
IV	34	17%
Grade		
A	35	18%
B	85	43%
C	80	40%
Extent		
Localized	122	61%
Generalized	78	39%
Clinical Attachment Loss		
1–2 mm	63	32%
3–4 mm	49	25%
5 mm and above	88	44%
Probing Depth		
4 mm and below	51	26%
5 mm	57	29%
6 mm and above	92	46%
Bone Loss Percentage		
%15 and below	61	31%
%15-%33	61	31%
%33 and above	78	39%
Number of teeth lost due to periodontitis		
No tooth loss	142	71%
1–4	23	11.5%
5 and above	35	17.5%
% Bone Loss/Age		
0.25 and below	64	32%
0.25–1	88	44%
1 and above	48	24%
Phenotype of destruction		
Heavy Biofilm deposits with low levels of destruction	74	37%
Destruction commensurate with biofilm deposits	117	58.5%
Destruction exceeds expectation given biofilm deposits	9	4.5%
Smoking		
Non-smoker	145	73%
≤ 9 cigarattes a day	20	10%
10 + cigarattes a day	35	18%
Diabetes		
No diabetes	170	85%
HbA1C7 ≤ 6.99%	14	7.0%
HbA1C7 ≥ 7%	16	8.0%
Plaque index; mean ± SD	1.94 ± 0.55	
Gingival index; mean ± SD	2.00 ± 0.47	

### Agreement between the RDs and the stage, grade, and extent of periodontitis determined by ChatGPT

ChatGPT correctly determined the stage in 59.5% of the cases, the grade in 50.5% of the cases, and the extent of periodontitis in 84.0% of the cases. The levels of agreement between the RDs and ChatGPT’s responses in terms of stage, grade, and extent of periodontitis were shown in Table 2. Moderate agreement was observed in terms of periodontitis stage (kappa of > 0.4 to < 0.6). Fair agreement was seen in terms of grade (kappa of > 0.2 to < 0.4). Regarding the extent, there was a substantial agreement (kappa of > 0.6 to < 0.8). ChatGPT used confident language consistently, even when incorrect (100%, 200 of 200).

**Table 2:** The agreement of ChatGPT's stage, grade and extent and RDs’ stage, grade and extent

RDs Stage					
	I *n* (%)	II *n* (%)	III *n* (%)	IV *n* (%)	Kappa
ChatGPT’s Stage					0.447
I	41 (91%)	31 (48%)	6 (11%)	0 (0%)	
II	4 (8.9%)	31 (48%)	8 (14%)	1 (2.9%)	
III	0 (0%)	2 (3.1%)	43 (75%)	29 (85%)	
IV	0 (0%)	0 (0%)	0 (0%)	4 (12%)	
RDs Grade					
	A *n* (%)	B *n* (%)	C *n* (%)		Kappa
ChatGPT’s Grade					0.284
A	33 (94%)	39 (46%)	19 (24%)		
B	2 (5.7%)	45 (53%)	38 (48%)		
C	0 (0%)	1 (1.2%)	23 (29%)		
RDs Extent					
	Localized, *n* (%)		Generalized, *n* (%)		Kappa
ChatGPT’s Extent					0.652
Localized	113 (93%)		23 (29%)		
Generalized	9 (7.4%)		55 (71%)		

Abbreviation: *RD* reference diagnose

^*^Cohen’s kappa test: The kappa values were interpreted as follows: ≤ 0 = poor agreement; 0–0.2 = slight agreement; 0.21–0.4 = fair agreement; 0.41–0.6 = moderate agreement; 0.61–0.8 = substantial agreement; and 0.81–1 = almost perfect agreement

### Correspondence among staging variables, RD’s stage and ChatGPT stage

**Fig. 4:**
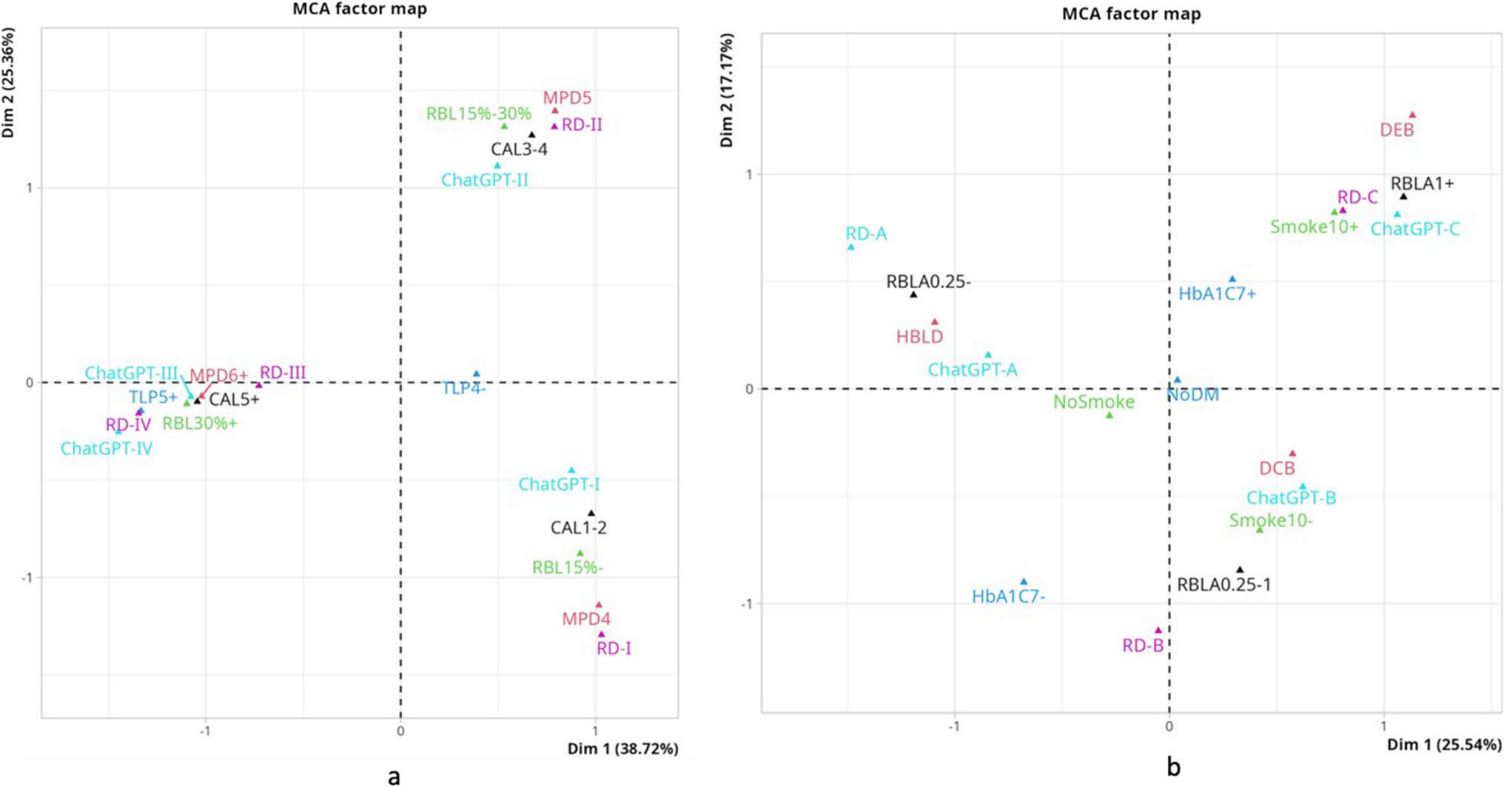
The multiple correspondence analysis between ChatGPT’s staging and the variables affecting the periodontitis stage (**a**); The multiple correspondence analysis between ChatGPT’s grading and the variables affecting the periodontitis grade (**b**). Abbreviations (a): MCA, multiple correspondence analysis; Dim, dimensions; ChatGPT-I, ChatGPT’s Stage I; ChatGPT-II, ChatGPT’s Stage II; ChatGPT-III, ChatGPT’s Stage III; ChatGPT-IV, ChatGPT’s Stage IV; RD-I, Reference Diagnoses’ Stage; RD-II, Reference Diagnoses’ Stage II; RD-III, Reference Diagnoses’ Stage III; RD-IV, Reference Diagnoses’ Stage IV; CAL, Clinical Attachment Loss; MPD, Maximum Probing Depth; RBL, Radiographic bone loss; TLP4-, The Number of Teeth Loss due to Periodontitis ≤ 4; TLP5 + , The Number of Teeth Loss due to Periodontitis ≥ 5. Abbreviations (b): MCA, multiple correspondence analysis; Dim, dimensions; ChatGPT-A, ChatGPT’s Grade A; ChatGPT-B, ChatGPT’s Grade B; ChatGPT-C, ChatGPT’s Grade C; RD-A, Reference Diagnoses’ Grade A; RD-B, Reference Diagnoses’ Grade B; RD-C, Reference Diagnoses’ Grade C; RBLA, Ratio of the percentage of radiographic bone loss to age; HBLD, Phenotype of destruction: heavy biofilm deposits with low levels of destruction; DCB, destruction commensurate with biofilm deposits; DEB, destruction exceeding expectations given the amount of biofilm deposits; Smok10 + , 10 ≤ cigarettes a day; Smok10-, ≤ 9 cigarettes a day; NoDM, No diabetes; HbA1C7-, Diabetes with HbA1c levels of < 7%; HbA1C7 + , Diabetes with HbA1c levels of ≥ 7%

The multiple correspondence analysis showed high variance (64.08%) between ChatGPT’s stage, RD’s stage and the variables affecting the periodontitis stage (CAL, PD, and RBL) (Fig. 4). There was observed correspondence among RD’s stage I, ChatGPT’s stage I, 1–2 mm CAL, PD of up to 4 mm and RBL below 15. A strong correspondence was seen among RD’s stage II, ChatGPT’s stage II, CAL of 3–4 mm, PD of up to 5 mm, and RBL between 15 and 30%. ChatGPT's stage IV, the number of teeth loss is five and above, ChatGPT's stage III, RD's Stage III, 5 mm and above CAL, 6 mm and above PD, RBL greater then 30% values were very close to each other. Additionally, they were located close to the point of origin. The variables and ChatGPT’s stages III-IV exhibited a lack of clear distinction.

### Correspondence among grading variables, RD’s grade and ChatGPT’s grade

In the context of multiple correspondence analysis, the proportion of variation accounted for by the variables ChatGPT's grade, RD's grade, RBL/Age, smoking and diabetes status, and the phenotype of destruction was found to be relatively low, namely at 42.71%. It was observed that ChatGPT’ grade B corresponds to DCB and 10 > cigarettes a day (Fig. 4). There was correspondence among ChatGPT’s Stage C, RD’s Stage C, RBL/Age ratio of > 1, 10 ≤ cigarettes a day and DEB but no correspondence was observed between ChatGPT’s Grade A-B and RD’s Grade A-B.

## Discussion

This study evaluated the ability of ChatGPT to classify periodontitis. This is the first study to evaluate the quality of information provided by a chatbot in the field of periodontology. The main findings of this study; (i) overall diagnosis was accurate in more than half of the cases; (ii) The accuracy of stage assignment (59.5%) and grade assignment (50.5%) may be considered reasonable. However, it is better in terms of extent and distribution (84.0%); (iii) There was moderate level agreement seen between the stage of ChatGPT and RD, however a fair level of agreement was found between the grade of ChatGPT and RD. In terms of the extent, a substantial agreement was reached; (iv) The language employed by ChatGPT exhibited a consistent usage of confident tone, even in instances where the periodontitis classification provided was found to be wrong (100%, 200 out of 200); (v) the lack of clear distinction was observed in the ChatGPT’s stage III-IV; (vi) the correspondence was noticed between the ChatGPT's Stage C and the RD's stage C, but no correspondence was found between ChatGPT's Grade A-B and RD's Grade A-B.

Conversational AI technology has the potential to be integrated into clinical practice, including periodontology. However, a notable limitation is the possibility of providing confident yet incorrect responses, known as hallucinations, especially in technical fields, such as medicine [21]. As these models gain popularity, it is important for them to handle ambiguous input effectively, especially when patients use them as online symptom checkers. Currently, most models, including ChatGPT, do not ask questions for clarification and instead proceed by assuming what the user means [9].

As this study is the first to evaluate the ability of ChatGPT to classify periodontitis, a comparison with similar studies cannot be made. However, a recent study found that ChatGPT’s answers to technical questions were of lower quality than answers to patients’ questions [22]. This result was consistent with ChatGPT's moderate performance in the classification of periodontitis, which is a technical characteristic. The results indicate that ChatGPT demonstrated a moderate level of agreement with the RDs in terms of the stage of periodontitis (kappa = 0.447). Additionally, there was substantial agreement between ChatGPT and the RDs in determining the extent of periodontitis (kappa = 0.652), while fair agreement was noted in terms of grade (kappa = 0.284).

In contrast with our study, Marini et al. found that the level of agreement between the standard diagnoses and dentists’ assessments was lower in terms of the extent of periodontitis than in terms of stage and grade [7]. The highest agreement in terms of extent observed in this study may have been due to the use of a single descriptive sentence that ChatGPT could interpret more easily, whereas the data used for grading and staging were inputted in longer sentences, which may have affected the results. In addition, the use of more objective data for staging, such as CAL, PD, and RBL, the interdependence of these data, and the independence of grade-modifying variables, such as smoking and diabetes, may have led to a higher level of agreement between ChatGPT and RDs in terms of stage than grade.

The highest agreement between RD and ChatGPT responses was observed in Stage I and Stage III. Considering that ChatGPT identifies the majority of cases as Stage I (39%) and Stage III (37%), this result is considered inevitable. However, it remains unclear why ChatGPT made such a definition.

Marini et al. reported that participants in their study experienced difficulties to discriminate between stages III and IV [7]. Similarly, ChatGPT was insufficient to discriminate between stages III and IV. Nevertheless, the high variance between ChatGPT’s staging, grading and the variables used to determine the stage and grade, presents potential for the application of ChatGPT in the classification of periodontitis.

Besides enhanced accuracy, using conversational AI models to support classification can provide further benefits. Like humans, AI can learn from past instances and improve diagnostic accuracy. This is achieved using a process known as reinforcement learning from human feedback, which is a reward model used to teach complex AI systems at scale without the need for extensive human supervision [23]. The more such models are used as diagnostic tools, the better they become at recognizing illnesses. Although ChatGPT was not designed for diagnostic purposes, as a conversation progresses with more specific information and ChatGPT corrects its mistakes, it may be able to provide more specific answers. However, such models can be sensitive to the phrasing used in the input. While the cases used in this study were well described, inadequate descriptions may lead to inaccurate results [9].

To enhance its classification accuracy, ChatGPT could be integrated into academic databases, such as the Web of Science, PubMed, or Scopus. However, the current version of ChatGPT is not suitable for academic use, as it lacks scientific reference–based responses. Also, the sources from which it retrieves medical information are not specified, making their accuracy and reliability uncertain. It is thus important to note that the medical information provided by ChatGPT is not academic. These limitations are expected to be overcome with the development of new model architectures and the use of larger training datasets [9]. Domain-specific AI language models are also being built for specific purposes. Examples are Galactica and DRAGON, which have demonstrated the utility of a curated scientific corpus of training data [24–26]. Such a model may also be developed specifically for periodontology.

This is the first study to evaluate the use of ChatGPT in periodontitis classification. However, this study also has several limitations. First, only English was used in the conversations with ChatGPT. The availability of ChatGPT in other languages also needs to be considered in periodontitis classification. One further limitation of our study is to the limited sample size of patients diagnosed with diabetes and individuals who smoke. The study's findings may have been impacted by the patient-reported data. Future studies may also examine whether ChatGPT’s classification ability can be enhanced by correcting its answers and providing information about the classification.

Tonetti et al. emphasize the importance of integrating additional information, including case phenotype, specific biomarkers, and the risk of systemic effects of periodontitis [10]. The classification of case phenotype, which describes the amount of biofilm deposits relative to periodontal destruction, remains relatively vague when used as indirect evidence to identify patients with high progression rates [17]. This lack of clarity constitutes a limitation of the study.

Another significant limitation of our study is the necessity for the bone loss data to be verbally described and input into the system by an external evaluator rather than being directly assessed from radiographic images. This limitation stems from the current version of the model, which cannot process and interpret medical images directly. However, it is important to note that advancements in AI, particularly in subsequent versions of ChatGPT, may address this shortcoming by incorporating the capability to analyze visual data such as radiographs. Future research should explore the efficacy of these advanced models in diagnosing periodontitis and other conditions directly from imaging data, potentially enhancing the role and importance of AI in clinical settings.

## Conclusion

ChatGPT was better at diagnosing the extent and distribution of periodontitis impacted by a single component than the stage and grade influenced by numerous components and modifying variables. ChatGPT's periodontitis classification performance was reasonable, but future improvements are expected. Further research is required to fully comprehend its capabilities and limitations and to identify optimal approaches to their integration into clinical practice. Nevertheless, while the use of AI models may increase, healthcare providers retain the ultimate responsibility for the final examination, diagnosis, and treatment decisions.
